# Supplementary material for: RNA editing generates cellular subsets with diverse sequence within populations
Source: Nat Commun. 2016 Jul 15;7:12145. doi: 10.1038/ncomms12145 (PMC4947178; doi:10.1038/ncomms12145)
Supplement: Supplementary Information — Supplementary Figures 1-6, Supplementary Tables 1-2, Supplementary Methods and Supplementary References [file ncomms12145-s1.pdf]

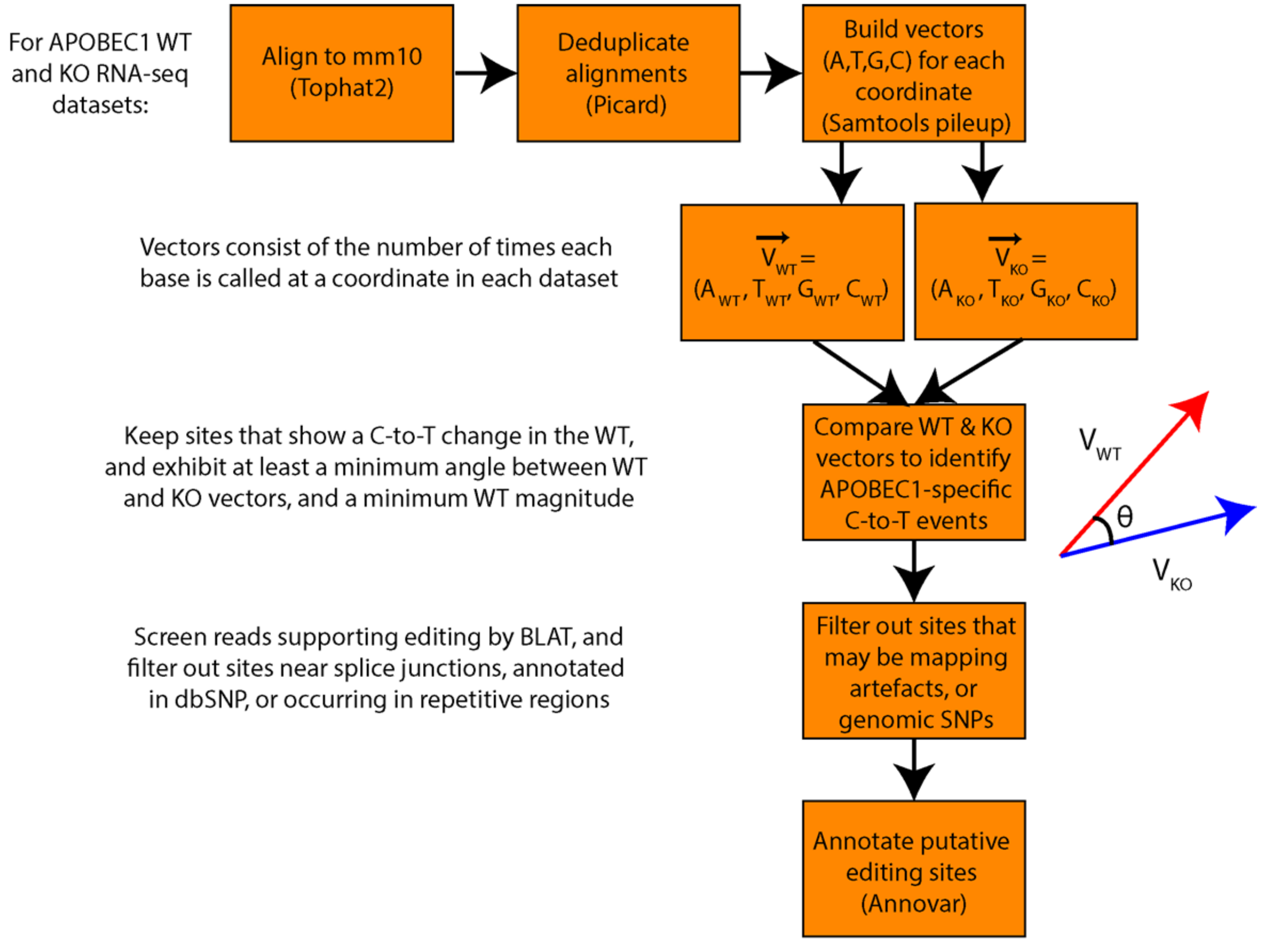

Supplementary Figure 1. RNA editing detection pipeline. Editing detection was performed by comparing APOBEC1 wildtype and *APOBEC1*$^{-/-}$ RNA-seq datasets for each cell type. Putative C-to-U edit sites occur in the Apobec1 wild-type sample, but not in the *APOBEC1*$^{-/-}$, and pass several stringent filters (see Methods for more details).

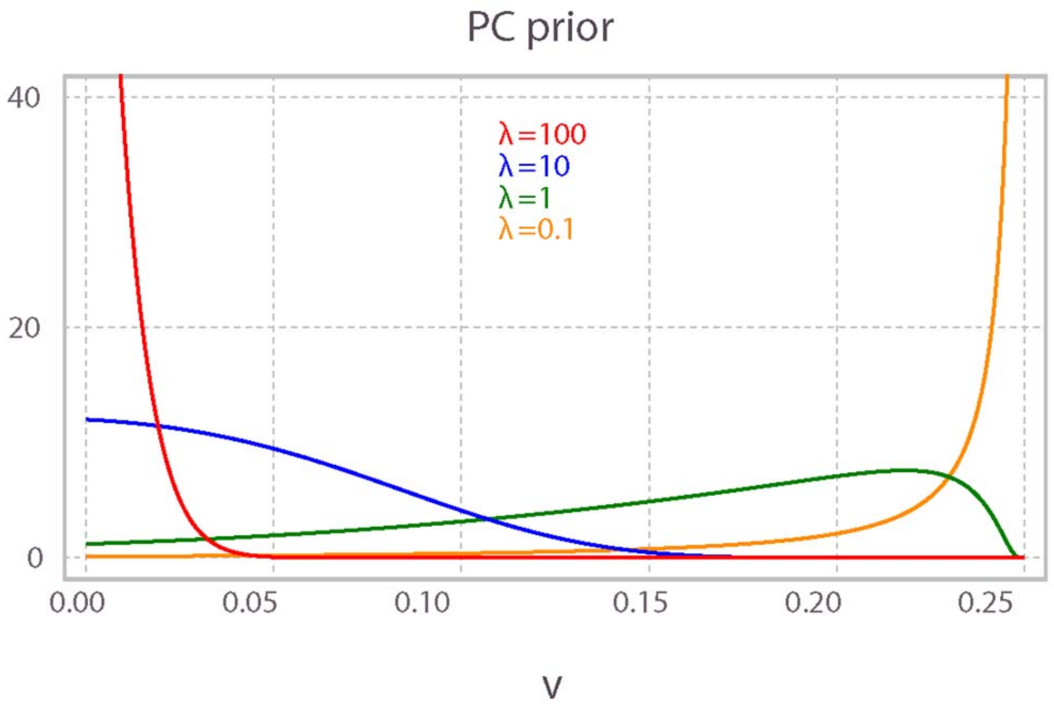

Supplementary Figure 2. Penalized complexity prior of the model for various values of its hyperparameter λ.

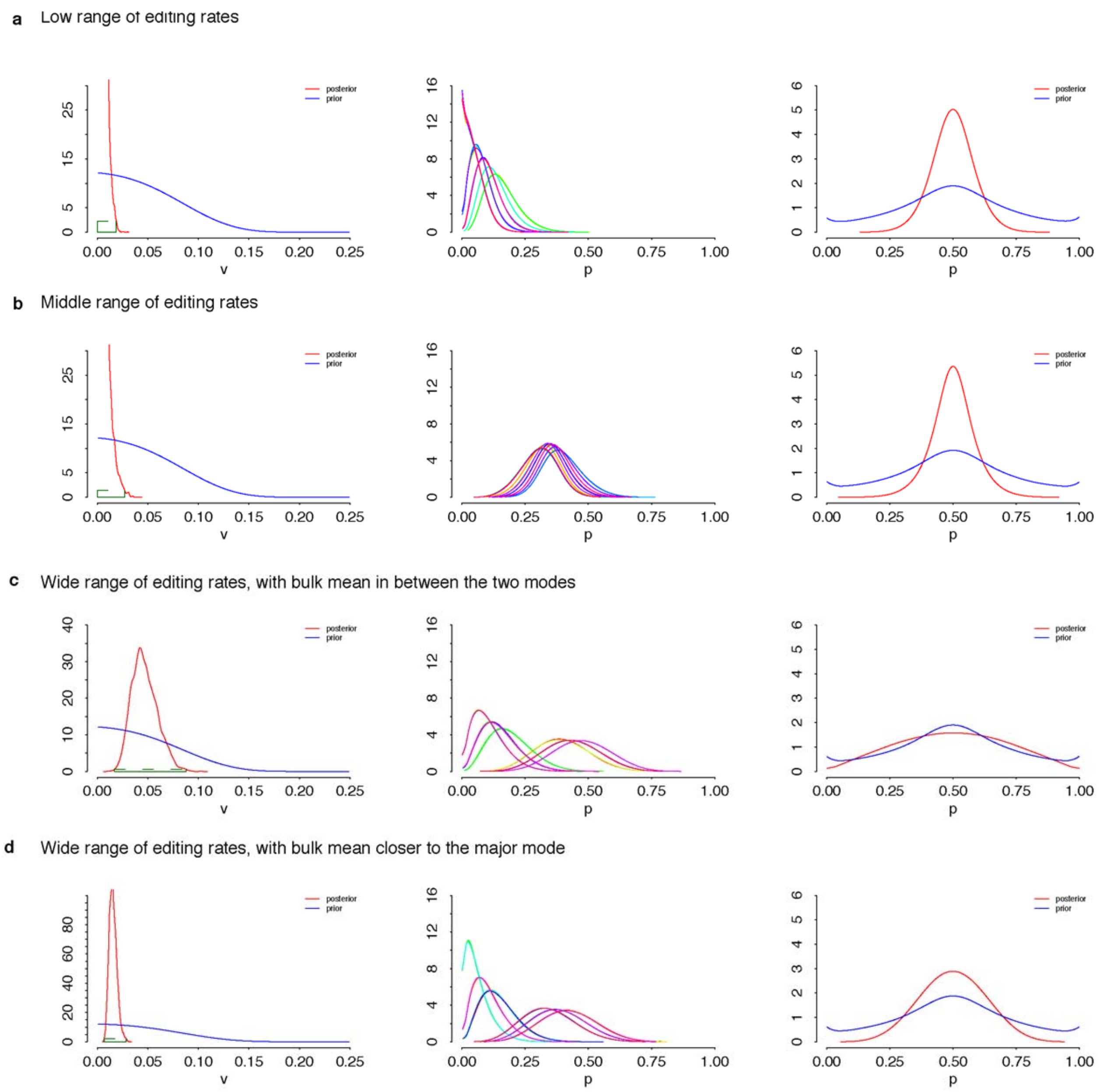

Supplementary Figure 3. Effect of varying ranges of editing rates on model. Histograms of variance of editing rates, editing rates and marginal editing rates for four artificial datasets, using editing rates within ranges we have observed from RNA-seq data. Each dataset consists of 20 cells with 20 reads per cell. (a) Low range of editing, with single cell editing rates ranging from 0 to 20%, and bulk editing rate set at 10%, (b) middle range of editing, with single cell editing rates ranging from 20-50%, and bulk editing rate set at 35%, (c) wide range of editing, with single cell editing rates ranging from 5-15% and 40-50%, with bulk editing rate set at 35%, and (d) wide range of editing, with single cell editing rates ranging from 5-15% and 40-50%, with bulk editing rate set at 10%.

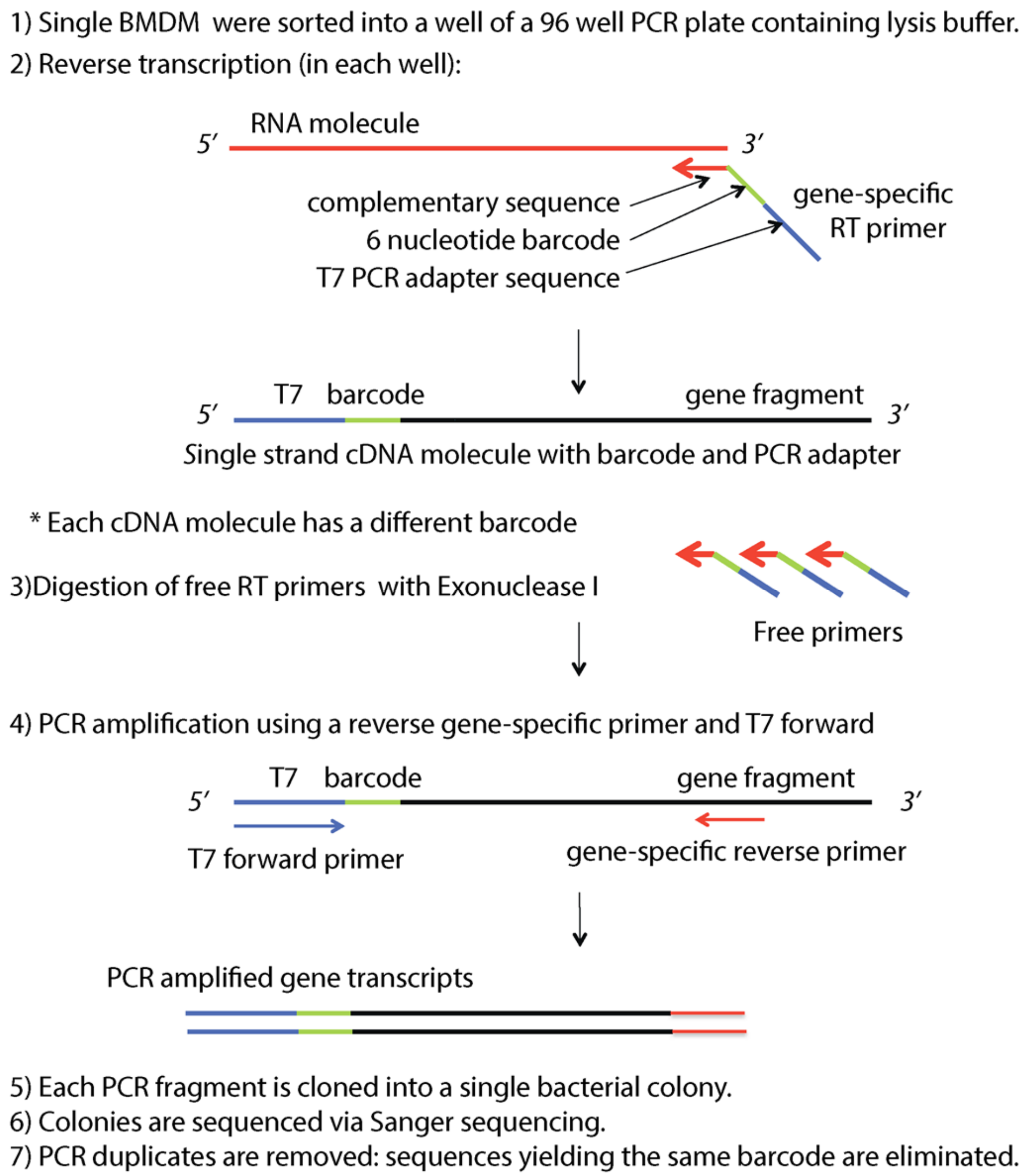

Supplementary Figure 4. Schematic of single cell sequencing with unique barcodes per transcript. Briefly, to analyze editing of specific sites in single cells, reverse-transcription/PCR amplification was done with gene specific primers and the OneStep RT-PCR kit (Qiagen), using a modified protocol. Single transcript molecules were tagged with barcoded gene-specific primers that have an additional universal sequence, used in reverse transcription. Primers were then digested with exonuclease I. Afterwards, a mix of universal forward and gene specific reverse primers were added to the PCR mix and 35 to 40 cycles of PCR were performed. The PCR products were introduced into bacteria using a TOPO TA cloning kit (Invitrogen) and single bacterial colonies were sequenced using Sanger sequencing. The resulting sequences were then aligned to the reference transcriptome (Macvector) and PCR duplicates were eliminated using the barcodes.

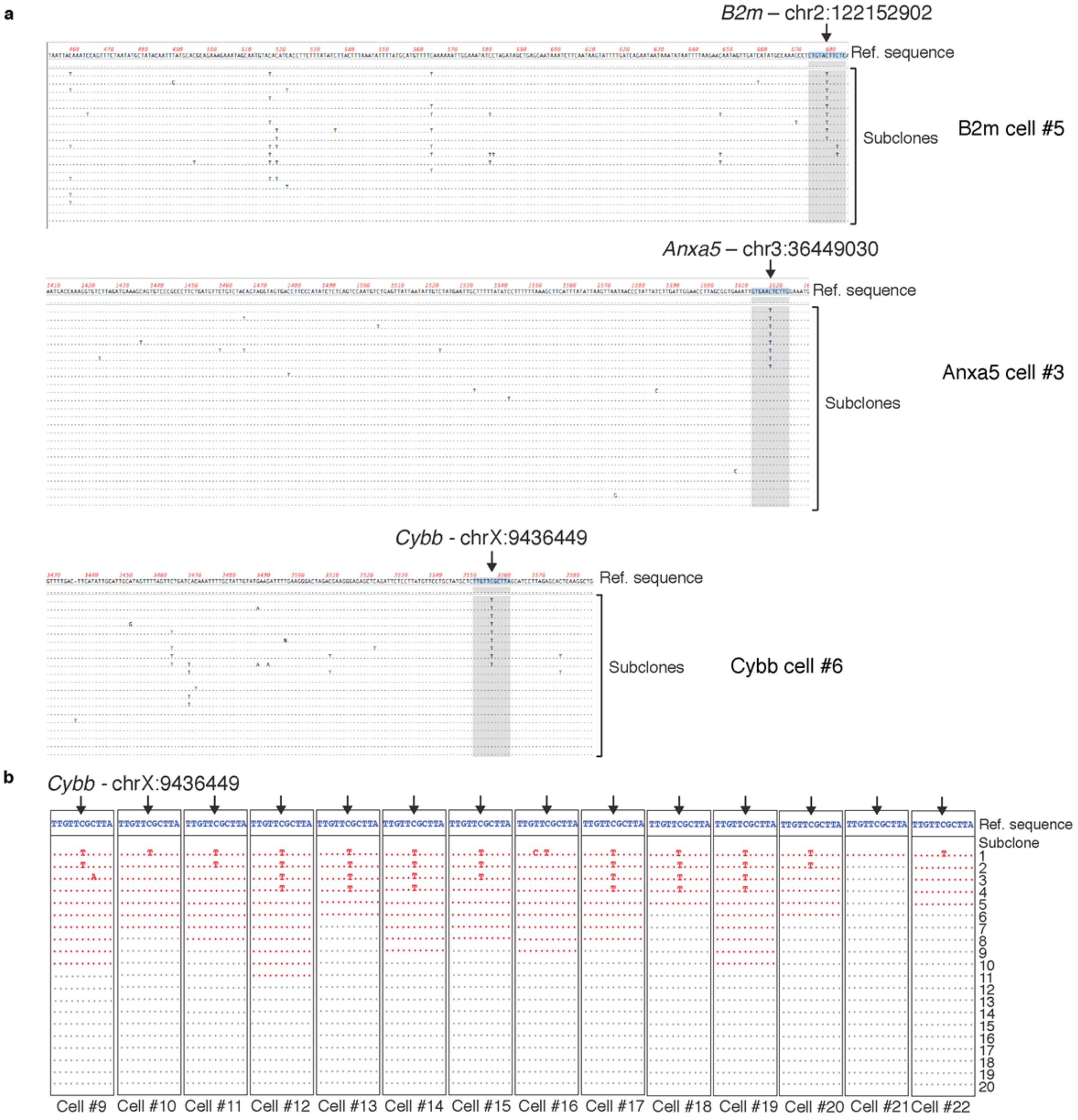

Supplementary Figure 5. Sanger sequencing of PCR amplicons from individual macrophages. (a) Amplicons (generated as indicated in Supplementary Fig. 4) display additional edited sites in transcripts outside of the predicted and simulated sites in B2m, Anxa5, and Cybb. (b) Amplicons from the Cybb transcript from additional single cells.

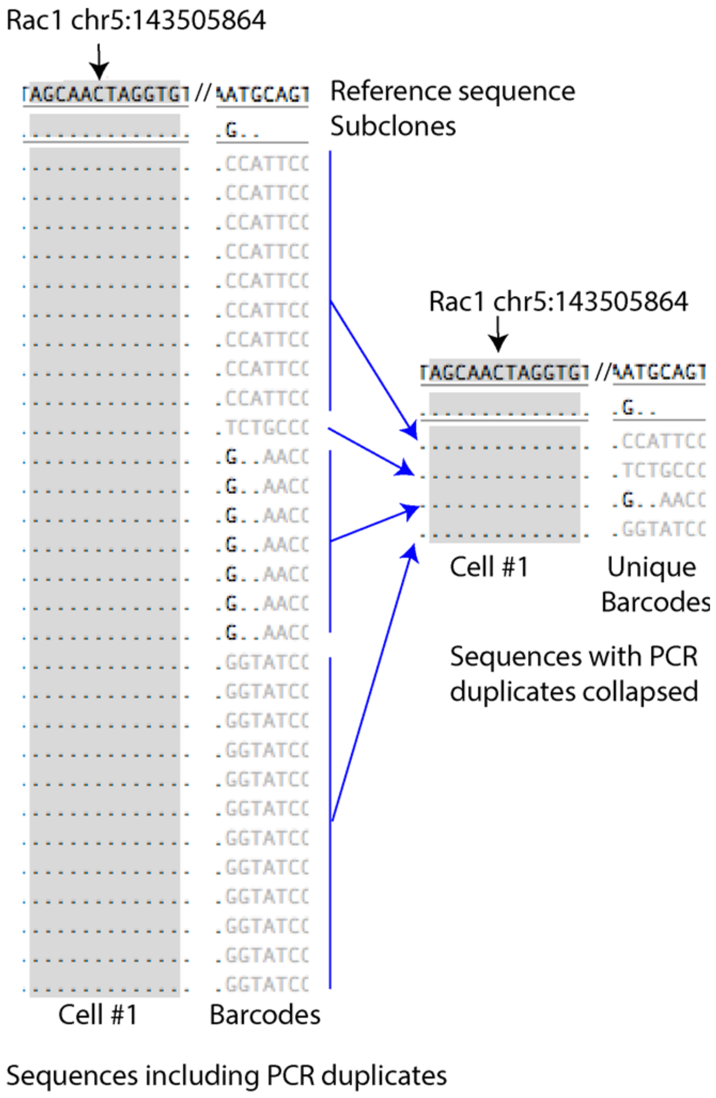

Supplementary Figure 6. Single cell PCR of lowly to moderately expressed transcripts reveals high rates of PCR duplication. Amplification of Rac1, a moderately expressed transcript, from a single cell reveals high rates of PCR duplication as indicated by the comparatively low rate of unique barcode sequences. The 29 sequences from cell #1 (left) collapsed to only 4 uniquely barcoded sequences (right). The percentage of unique barcodes from Rac1 amplicons across the 15 cells assayed is tabulated in Supplementary Table 2.

Supplementary Table 1. RNA editing detection pipeline performance. The number of hits as a function of filtering parameters for bulk, PCR-deduplicated RNA-seq data. With the vector approach, putative hits are filtered by a minimum magnitude ("mag", of the vector containing the putative edit) and a minimum angle ("ang", in radians) between the wildtype and *APOBEC1*$^{-/-}$ vectors at the same coordinate. Inverse hits are the number of hits yielded when treating the *APOBEC1*$^{-/-}$ dataset as the wildtype, and the wildtype as the *APOBEC1*$^{-/-}$. The inferred false positive rate (FPR) is calculated as the number of inverse hits divided by the number of hits called with the correct comparison.

Filters	Hits	%UTR3	Inverse hits	Inferred FPR
**12mag,0.11ang**	510	95%	16	3%
**12mag,0.24ang**	56	95%	1	2%
**15mag,0.11ang**	410	96%	11	3%
**15mag,0.24ang**	47	98%	1	2%
**7.2mag,0.11ang**	662	94%	22	3%
**No mag or angle filter**	1752	94%	78	4%

Supplementary Table 2. Percentage of uniquely-barcoded sequences obtained from Rac1 amplicons derived from single macrophages.

Cell #	Unique barcode %
1	13%
2	20%
3	37%
4	20%
5	23%
6	23%
7	33%
8	40%
9	26%
10	33%
11	50%
12	20%
13	37%
14	23%
15	10%

Supplementary Methods

Hierarchical Model for Editing Rates

Denote by $x_{i,j} \in \mathbb{N}_0 = \mathbb{N} \cup \{0\}$ the number of edited reads that were measured at genomic coordinate i in specific cell j, and denote by $n_{i,j} \in \mathbb{N}_0$ the corresponding total number of mapped reads (edited and unedited). We begin with two basic assumptions:

(A1) The number $n_{i,j}$ of total mapped reads does not depend on the editing rate $p_{i,j}$.

(A2) The event that a particular read corresponds to an edited sequence is statistically independent of the same event for all other reads, conditional upon the edit rate $p_{i,j}$.

With (A1), we assume that RNA-seq sampling of the transcriptome is unbiased, in that transcript detection is independent of sequence. While ideally detection of transcripts with RNA-seq should be agnostic of sequence, there are in actuality known biases to 3' ends of transcripts with poly-A enrichment, for instance, and with GC-content[1]. The mappability of a region also affects measurement (if we can't unambiguously map a read to a region, it isn't counted). But since we expect reads containing an edit versus an unedited sequence from the same transcript to be sampled at equal frequency, this assumption holds for our purposes. (A2) is an "exchangeability" assumption[2], which is satisfied if no transcripts are counted more than once. Since we remove PCR duplicates from alignments, we expect this assumption to be fulfilled. Under these assumptions the first component of our hierarchical model is a binomial distribution (for the number of edited reads):

$$x_{i,j} | p_{i,j}, n_{i,j} \sim \text{Bin}(n_{i,j}, p_{i,j}) \quad (1)$$

for each $i = 1,2,...,I$ and $j = 1,2,...,J$.

The second component of the hierarchy is used to couple together the different edit rate parameters $\{p_{i,j}\}$. Denote by $x_i \in \mathbb{N}_0$ the number of edited reads that were measured at genome coordinate i in the bulk RNA-seq experiment, and denote by $n_i \in \mathbb{N}_0$ the corresponding total number of mapped reads. We then assume the following:

(A3) The edit rate $p_i \in [0, 1]$ for the bulk experiment is approximately equal to x_i/n_i.□

(A4) Our J single cells can be considered to be drawn independently at random from the population of J' cells comprising the bulk experiment, where $J' >> J$.

(A3) is satisfied if the number of total reads n_i from the bulk experiment associated with site i is sufficiently large. (A4) supposes that the J single cells that we consider are "typical", in a precise statistical sense. The combined effect of (A3) and (A4) is to place a hard statistical constraint on our hierarchical formulation, namely that

$$\mathbb{E}[p_{i,j}] = p_i = \frac{x_i}{n_i}, \quad (2)$$

where the randomness in $p_{i,j}$ occurs through the choice of the single cell j from the population (J' possibilities). Such a constraint could be highly informative and a careful statistical analysis should therefore take this into account. However this class of problem does not appear to have been considered in the existing binomial regression literature, where p_i would essentially be treated as an unknown parameter to be estimated. The remainder of this section details a novel modelling approach that accounts for the constraint in Equation (2).

Denote by $v_i \in [0, p_i(1-p_i)]$ the variance of the editing rates at location i across the J' cells that were involved in the bulk experiment. Information sharing among the J editing rates $\{p_{i,j}\}$ in our (non-standard) setting was facilitated using a beta probability distribution

$$p_{i,j} \mid p_i, v_i \sim \text{Beta}^*(p_i, v_i). \qquad (3)$$

Here $\text{Beta}^*(p, v)$ is the standard beta distribution $\text{Beta}(a, b)$, where $a, b > 0$, reparametrized in the mean-variance parameterization; i.e.

$$P(p; a, b) = \frac{p^{a-1}(1-p)^{b-1}}{B(a,b)}, \qquad (4)$$

where $B(a, b)$ is the Beta function and

$$a = \left[\frac{p(1-p)}{v} - 1\right] p, \quad b = \left[\frac{p(1-p)}{v} - 1\right](1-p). \qquad (5)$$

From Equation (2) we are able to fix $p_i = x_i / n_i$ and thus Equation (5) represents a parametric statistical model with one degree of freedom. Denote this distribution by $B(v_i)$, where the dependence on p_i (assumed known) is suppressed for clarity. The remaining unknown parameter v_i is highly interpretable; indeed this is the quantity of scientific interest, with $v_i = 0$ corresponding to identical editing rates across the J single cells and $v_i = p_i(1-p_i)$ corresponding to statistically independent $p_{i,j} \mid p_i, v_i \sim \text{Uniform}(0, 1)$ error rates across the J single cells.

The statistical specification is completed with one final assumption:

(A5) The edited sites indexed by i (and measurements thereof) can be treated independently.

(A5) improves interpretability and permits us to analyze each genomic location on an individual basis. Since there is evidence that RNA editing can occur processively along transcripts[3], there is in actuality correlation in the editing rates across edited genomic coordinates i that occur on the same gene. However it is far from clear how such association should be encoded into inference and we leave this as an open direction for further research.

Bayesian Inference. The previous section specified a hierarchical statistical model such that, for each location i, the model is parametrized by the edit rates $p_{i,1}, \ldots, p_{i,J}$ and their variance v_i. Under (A5) we can restrict attention to just a single genomic location i; in this section we leave the i index implicit to improve clarity of the notation. Below we discuss how to estimate

parameters $\theta = \{ p_1, \ldots, p_J, v\}$ from their associated data $D = \{(x_j, n_j)\}$. (Data from the bulk experiment are assumed fixed and known throughout.)

Given that our analysis is primarily exploratory, we elected to follow the Bayesian paradigm and we focus here on posterior inference for the model parameters θ. In brief, the Bayesian approach begins by placing a prior probability distribution

$$P(\theta) = P(p_1, \ldots, p_J | v)\, P(v) \tag{6}$$

over model parameters and then applying Bayes' rule

$$P(\theta|D) = \frac{P(D|\theta)P(\theta)}{P(D)} \tag{7}$$

in order to obtain the posterior distribution $P(\theta|D)$. Formally the prior distribution $P(\theta)$ encodes our (possibly subjective) *a priori* uncertainty with respect to the model parameters. The first term in Equation (6) is simply the beta density that was specified earlier in Equation (3), whilst the second term $P(v)$ requires elicitation. In this paper we take a Bayesian approach to prior elicitation.

Derivation of the PC Prior. PC priors can be described as a fusion between conventional subjective Bayesian analysis and objective Bayesian techniques[4], meaning that they aim to provide useful statistical regularization in the absence of subjective prior knowledge by providing a semi-automatic approach to prior construction in hierarchical models. PC priors specifically are predicated on the mantra that simpler "base" models should be preferred to more complex alternatives. PC priors, recently proposed by[5], have a natural connection to the well-known Jeffreys' priors[6] in the sense that they are invariant to reparameterizations. However, compared to Jeffrey's priors there is a more explicit connection with Occam's razor that fits elegantly with the scientific null hypothesis ($v = 0$) that we investigate in this work. Below we construct a PC prior $p(v)$ for the unknown variance parameter.

A PC prior is defined by explicitly stating a "base model" $\mathcal{M}$, with respect to which $B(v)$ is a more complex alternative. For us, the base model $\mathcal{M}$ is the $\mathcal{B}(v_0)$ distribution where $v_0 = 0$. i.e. there is no randomness in the probability model. This coincides with the scientific null hypothesis $v = 0$ and captures Occam's principle. Then the Kullback-Leibler divergence $KL(\mathcal{B}(v)||\mathcal{M})$ between these distributions is computed. Define the (pseudo-)distance

$$d(v) = \sqrt{2KL(\mathcal{B}(v)||\mathcal{M})}$$

Finally the PC prior is defined by placing an exponential distribution $P(d) = \lambda e^{-\lambda d}$ on the distance d. This explicitly penalizes model complexity, quantified in terms of distance to the base model. The PC prior density follows via the chain rule:

$$P(v) = \lambda e^{-\lambda d(v)} \left|\frac{\partial d(v)}{\partial v}\right|$$

Since the Kullback-Leibler distance is parametrization invariant, it follows that so is the PC prior. Below we derive a closed-form expression for $P(v)$.

Begin by noting that the Kullback-Leibler divergence between two beta densities Beta(a, b), Beta(a_0,b_0), in the standard parametrization, is given by

$$\log\left(\frac{B(a_0,b_0)}{B(a,b)}\right) + (a - a_0)\psi(a) + (b - b_0)\psi(b) + (a_0 - a + b_0 - b)\psi(a + b). \quad (8)$$

Here $\psi(y) = \frac{d\log\Gamma(y)}{dy}$ denotes the digamma function, the derivative of the gamma function. Next we aim to take the limit $v_0 \rightarrow 0$ in Equation (8), corresponding to a_0, $b_0 \rightarrow \infty$. In this limit we can appeal to Sterling's approximation to obtain

$$B(a_0, b_0) \sim \frac{\sqrt{2\pi} a_0^{a_0 - \frac{1}{2}} b_0^{b_0 - \frac{1}{2}}}{(a_0 + b_0)^{a_0 + b_0 - \frac{1}{2}}}. \quad (9)$$

This implies that asymptotically

$$KL(\text{Beta}(a, b)||\text{Beta}(a_0, b_0)) \sim a_0 \log(a_0) + b_0 \log(b_0) - (a_0 + b_0)\log(a_0 + b_0)$$
$$-a_0\psi(a) - b_0\psi(b) + (a_0 + b_0)\psi(a + b). \quad (10)$$

From the definition of a_0 and b_0 in terms of p and v_0, given in (5), we obtain

$$KL(\text{Beta}(a, b)||\text{Beta}(a_0, b_0)) \sim \frac{p(1-p)}{v_0}[\psi(a + b) - p\psi(a) - (1 - p)\psi(b)] \quad (11)$$

and hence

$$d(v) \sim \left\{\frac{2p(1-p)}{v_0}[\psi(a + b) - p\psi(a) - (1 - p)\psi(b)]\right\}^{1/2}. \quad (12)$$

Finally we assume an exponential distribution where

$$P(d) = \begin{cases} C\lambda e^{-\lambda d} \text{ whenever } 0 < d < d_{max} \\ 0 \text{ otherwise} \end{cases} \quad (13)$$

where $C^{-1} = \int_0^{d_{max}} \lambda e^{-\lambda y}\, dy$ is a normalising constant resulting from the truncation of the exponential distribution, $d_{max} = max_{v\in V}\, d(v)$ and

$$\lambda \sim \left\{\frac{v_0}{2p(1-p)}\right\}^{1/2}. \quad (14)$$

The PC prior with hyper-parameter λ is given by

$$P(v) \sim \lambda\exp\{-\lambda[\psi(a + b) - p\psi(a) - (1 - p)\psi(b)]\}^{1/2} \quad (15)$$

$$\times \left| \underbrace{\frac{\partial}{\partial v}[\psi(a+b) - p\psi(a) - (1-p)\psi(b)]^{1/2}}_{(*)} \right|$$

for all $v \in V$ and $P(v) = 0$ otherwise. The term $(*)$ can be obtained using the chain rule:

$$(*) = \frac{p(1-p)\left|p^2\psi_1(a) + (1-p)^2\psi_1(b) - \psi_1(a+b)\right|}{2v^2[\psi(a+b) - p\psi(a) - (1-p)\psi(b)]^{\frac{1}{2}}} \tag{16}$$

where $\psi_1(y) = \frac{d\psi(y)}{dy} = \frac{d^2 \log\Gamma(y)}{dy^2}$ is the trigamma function. The PC prior derived above is fully specified up to one hyper-parameter λ. Supplementary Fig. 2 displays the PC prior for a range of values of its hyper-parameter λ.

Following the recommendation in [5] we set λ subjectively in terms of $\mathbb{P}[v > v_L] < 0.01$. The quantity v_L corresponds to the level of variability in the editing rates $p_1,...,p_J$ would be considered *a priori* unlikely at the 1% level. Results in this paper were based on a conservative prior that assumes little variability ($v_L = 0.142$).

Computational Implementation. Bayesian inference is based on the posterior distribution of parameters θ given data D, however the associated probability density function $P(\theta|D)$ in our case is not available in closed-form. We therefore employed standard sampling techniques based on Markov chain Monte Carlo (MCMC)[7] to obtain an empirical approximation to the posterior distribution that becomes exact in the ergodic limit. Efficient MCMC proposals were constructed by exploiting beta-binomial conjugacy, since the joint probability density

$$P(\{p_j\}, v, \{x_j\}|\{n_j\}) = P(v)P(\{p_j\}|v)P(\{x_j\}|\{p_j\}, v) \tag{17}$$

$$= P(v)\prod_{j=1}^{j} \text{Beta}^*(p_j; p, v)\text{Bin}(x_j; n_j, p_j) \tag{18}$$

implies that

$$P(\{p_j\}, v|\{x_j\}|\{n_j\}) \propto P(v)\prod_{j=1}^{j} \text{Beta}(p_j; a + x_j, b + n_j - x_j) \tag{19}$$

where a, b are the beta distribution parameters corresponding to p, v in the mean-variance parameterization. Here we write $\text{Beta}(p_j\,; a + x_j, b + n_j - x_j)$ to denote the probability density function of a beta distribution with parameters $a + x_j$, $b + n_j - x_j$, evaluated at the location p_j, with similar notation used in the transformed beta and binomial cases. It follows that we can construct an efficient Metropolis-within-Gibbs algorithm where $\{p_j\}|v$ can be sampled exactly and $v|\{p_j\}$ requires a Metropolis-Hastings step. For the Metropolis-Hastings step we employed the logit-transform

$$v \mapsto \text{logit}\left(\frac{v}{p(1-p)}\right) \tag{20}$$

to provide support for the unnormalized distribution of $v|\{p_j\}$ over the real line. In particular, the random adaptive Metropolis (RAM) sampler was used to sample from $v|\{p_j\}$, see[8].

For each reported simulation, 11,000 Gibbs iterations were run, of which the first 1,000 were discarded as burn-in. At each Gibbs step, a single RAM iteration was run in order to exploit the Beta-Binomial conjugacy of the second Gibbs step.

Development of credible intervals. We formulate a classification rule in order to decide whether a genomic location is likely to display variable editing rates or otherwise, based on our available data D. For this purpose we chose to consider the $100(1-\alpha)\%$ highest posterior density (HPD) credible interval

$$I_{\alpha,i}(D) = \{0 \le v_i \le p_i(1-p_i) \text{s.t.} P(v|D) > \delta_i\}, \quad (21)$$

defined such that δ_i is the largest number satisfying

$$\int_{I_{\alpha,i}(D)} P(v|D)dv = 1 - \alpha, \quad (22)$$

where $P(v|D)$ is the posterior of v.

A classification rule is then constructed according to

$$r_{\alpha,i}(D) = \begin{cases} 0 \text{ if } 0 \in \mathrm{I}_{\alpha,i}(\mathrm{D}) \\ 1 \text{ otherwise} \end{cases} \quad (23)$$

where $r_{\alpha,i}(D) = 1$ corresponds to variable editing rates and $r_{\alpha,i}(D) = 0$ corresponds to non-variable editing rates. That is, we classify a genomic location i as a location with variable editing rate when the $100(1-\alpha)\%$ HPD credible interval does not contain the base model $v = 0$.

In practice, the credible interval of v has been approximated via an iterative algorithm. For successive increments of δ_i, the algorithm alternates between numerical root finding of $P(v|D) = \delta_i$ with respect to v and numerical integration of the integral on the right-hand side of (22). The algorithm stops after reaching a pre-defined level of relative tolerance.

Supplementary References

1. Cheung, M.-S., Down, T. A., Latorre, I. & Ahringer, J. Systematic bias in high-throughput sequencing data and its correction by BEADS. *Nucleic Acids Res.* **39,** e103 (2011).
2. Leonard, T. Bayesian methods for binomial data. *Biometrika* **59,** 581–589 (1972).
3. Carmi, S., Borukhov, I. & Levanon, E. Y. Identification of widespread ultra-edited human RNAs. *PLoS Genet.* **7,** e1002317 (2011).
4. Berger, J. The case for objective Bayesian analysis. *Bayesian Anal.* **1,** 385–402 (2006).
5. Simpson, D. P., Rue, H., Martins, T. G., Riebler, A. & Sørbye, S. H. Penalising model component complexity: A principled, practical approach to constructing priors. *arXiv:1403.4630 [stat]* (2014).
6. Jeffreys, H. An Invariant Form for the Prior Probability in Estimation Problems. *Proceedings of the Royal Society of London A: Mathematical, Physical and Engineering Sciences* **186,** 453–461 (1946).
7. Gilks, W. R. in *Encyclopedia of Biostatistics* (John Wiley & Sons, Ltd, 2005).
8. Haario, H., Saksman, E. & Tamminen, J. An adaptive Metropolis algorithm. *Bernoulli* **7,** 223–242 (2001).
